# Complete genome sequence of a novel avian paramyxovirus isolated from wild birds in South Korea

**DOI:** 10.1007/s00705-017-3588-6

**Published:** 2017-10-16

**Authors:** Jipseol Jeong, Youngsik Kim, Injung An, Seung-Jun Wang, Yongkwan Kim, Hyun-Jeong Lee, Kang-Seuk Choi, Se-Pyeong Im, Wongi Min, Jae-Ku Oem, Weonhwa Jheong

**Affiliations:** 1 Environmental Health Research Department, National Institute of Environmental Research, Hwangyeong-ro42, Seo-gu, Incheon, 22689 Republic of Korea; 2 Avian Disease Research Division, Animal and Plant Quarantine Agency, 177 Hyeokshin 8-ro, Gimcheon, Republic of Korea; 3 College of Veterinary Medicine & Institute of Animal Medicine, Gyeongsang National University, Jinju, 52828 Republic of Korea; 4 Department of Veterinary Infectious Diseases, College of Veterinary Medicine, Chonbuk National University, Iksan, Republic of Korea

**Keywords:** Wild birds, Avian paramyxovirus (APMV), APMV-17, South Korea

## Abstract

A novel avian paramyxovirus (APMV), Cheonsu1510, was isolated from wild bird feces in South Korea and serologically and genetically characterized. In hemagglutination inhibition tests, antiserum against Cheonsu1510 showed low reactivity with other APMVs and *vice versa*. The complete genome of Cheonsu1510 comprised 15,408 nucleotides, contained six open reading frames (3’-N-P-M-F-HN-L-5’), and showed low sequence identity to other APMVs (< 63%) and a unique genomic composition. Phylogenetic analysis revealed that Cheonsu1510 was related to but distinct from APMV-1, -9, and -15. These results suggest that Cheonsu1510 represents a new APMV serotype, APMV-17.

**Electronic supplementary material:**

The online version of this article (doi:10.1007/s00705-017-3588-6) contains supplementary material, which is available to authorized users.

Avian paramyxoviruses (APMVs) are negative-sense single-stranded RNA viruses that belong to the genus *Avulavirus* within the family *Paramyxoviridae.* To date, at least 16 serotypes (APMV-1 to APMV-14, and two named APMV-15) have been recognized [2, 4, 7, 8, 11–14]. The genomes of APMVs range from 14.9 to 17.4 kb in length and encode at least six structural proteins: nucleocapsid (N), phosphoprotein (P), matrix (M), fusion (F), hemagglutinin-neuraminidase (HN), and large polymerase (L). Two additional proteins, V and W, may be produced by an RNA editing event during transcription of the P gene [2].

APMV-1 to -9 had been isolated from chickens, ducks, turkeys, and wild birds all over the world prior to the 1980s [2]. Of these serotypes, APMV-1, also known as Newcastle disease virus, is well characterized because of its high lethality in poultry [1]. APMV-2, 3, 6, and 7 are also associated with respiratory illness in chickens and turkeys. APMV-4, 8, and 9 have been mainly isolated from waterfowl, such as ducks and geese, whereas APMV-5 was isolated from budgerigars, in which it causes fatal disease [1, 6, 9]. Following the emergence and spread of highly pathogenic avian influenza (HPAI) in the early 2000s, extensive avian influenza surveillance programs in aquatic wild birds around the globe have expanded our knowledge of the ecology of APMV. In addition to the nine previously known APMV serotypes, seven novel APMV serotypes have been isolated from wild bird populations worldwide during AI surveillance [4, 7, 8, 11–14]. APMV-10, -11, -12, -13, and -14 were isolated from rockhopper penguins (*Eudyptes chrysocome*), common snipe (*Gallinago gallinago*), Eurasian wigeon (*Anas penelope*), migratory geese, and ducks, respectively. Most recently, two different putative APMV-15s were identified in migratory birds in South Korea (APMV-15(Kr)) and Brazil (APMV-15(Br)), one of which should be re-annotated as APMV-16 [7, 13].

The Ministry of Environment has conducted an AI surveillance program of migratory birds in South Korea since 2012. On January 7, 2015, a viral agent with hemagglutination activity was isolated from a single fecal sample collected from a migratory bird habitat in Cheonsu-bay, in the western region of South Korea (GPS coordinates 36°36′52.84″ N, 126°29′12.31″ E), but it was not an AI virus. In this study, we provide the first description of the serological and genomic features of this novel APMV virus, APMV/wild birds/Cheonsu1510/2015 (Cheonsu1510).

To determine the serotype of the unidentified APMV, a cross-hemagglutination-inhibition (HI) test was performed using a reference panel comprising antigens and chicken antisera against representatives of APMV-1 to APMV-9 (except APMV-5; National Veterinary Service Laboratories, USA) using the method outlined in the Terrestrial Manual of the World Organisation for Animal Health (OIE) [5]. Chicken antiserum against Cheonsu1510 was prepared by intravenously injecting 3-week-old specific pathogen-free (SPF) chickens with purified Cheonsu1510 (10^9.0^ 50% egg infectious dose [EID_50_] per dose) as described previously [7].

Viruses propagated in 9- to 11-day-old SPF embryonated chicken eggs were used for total RNA extraction using TRIzol Reagent (Life Technologies, USA). The nucleotide (nt) sequence of the full viral genome was determined using a next-generation sequencing (NGS) approach. The cDNA libraries were prepared for 100-bp paired-end sequencing using a TruSeq RNA Sample Preparation Kit (Illumina, CA, USA) [3]. Briefly, mRNAs were purified and fragmented from 2 μg of total RNA using oligo (dT) magnetic beads and used as a template for cDNA synthesis through random hexamer priming. Paired-end sequencing was performed using an Illumina HiSeq2500 System (Illumina). The reads of the full-length viral genome were assembled *de novo* with SPAdes assembler version 3.7 [3]. To confirm the NGS results, a conventional reverse transcription polymerase chain reaction (RT-PCR) (iNtRon Biotechnology, Seoul, Korea) was performed with virus-specific primers (sequences available on request). PCR-amplified segments were purified using a QIAquick PCR Purification Kit (QIAGEN, Hilden, Germany) and sequenced directly by the Sanger method at Macrogen, Korea. The sequence obtained by the Sanger method was 100% identical to that obtained by NGS sequencing. Nucleotide sequences were deposited in the GenBank database (accession no. MF594598).

Sequences were analyzed using BLAST (National Center for Biotechnology Information, USA) to identify related sequences and aligned using CLUSTALW2. Multiple alignments were used to infer the phylogenies, with the maximum-likelihood (ML) method implemented in MEGA 6. To obtain the ML tree topologies, 1,000 bootstrap replicates were performed for each dataset. The inferred tree topologies were inspected visually using FigTree version 1.3.1.

The antiserum titers against each representative APMV serotype were highest with the homologous viruses (Table 1). The HI titer of antiserum against isolated Cheonsu1510 had the highest HI with the homologous virus (1:128) and reacted only weakly with APMV-1 (1:16) and -9 (1:8). The HI titers of the Cheonsu1510 isolate with antisera against the APMVs were less than 1:16 for all except APMV-9 (1:32). The Cheonsu1510 virus showed an R-value < 0.05 to other APMVs except for APMV-1 (0.063; Online Resource 1).

**Table 1:** Results of cross-haemagglutination-inhibition tests with representative APMVs and homologous chicken antisera

Antigen	Antiserum to								
	Cheonsu 15-10	APMV-1	APMV-2	APMV-3	APMV-4	APMV-6	APMV-7	APMV-8	APMV-9
Cheonsu1510	**128**	16	< 2	< 2	< 2	< 2	16	8	32
APMV-1	16	**512**	< 2	8	< 2	< 2	32	4	32
APMV-2	< 2	< 2	**512**	4	4	16	32	16	16
APMV-3	4	16	4	**256**	< 2	< 2	64	16	32
APMV-4	< 2	8	< 2	< 2	**16**	< 2	16	< 2	16
APMV-6	< 2	< 2	8	4	< 2	**128**	16	4	4
APMV-7	< 2	4	4	4	< 2	< 2	**512**	8	8
APMV-8	4	8	8	8	4	4	32	**1024**	16
APMV-9	8	16	< 2	4	< 2	< 2	32	4	**1024**

A cross-haemagglutination-inhibition (HI) test was performed using a reference panel comprising antigens and chicken anti-sera against representatives of APMV-1 to APMV-9 (except APMV-5; National Veterinary Service Laboratories, USA) using the method outlined in the World Organization for Animal Health (OIE) Terrestrial Manual [5]

The complete genome of Cheonsu1510 consisted of 15,408 nt, and making it closest in size to APMV-9, at 15,438 nt (Fig. 1). The genomic organization of the virus was typical of the genus *Avulavirus*, with six genes (3’-leader-N-P-M-F-HN-L-trailer-5’), including intergenic regions of 10–35 nt. Theoretical amino acid (aa) lengths of the putative proteins were as follows: N, 487 aa; P, 439 aa; M, 365 aa; F, 551 aa; HN, 567 aa; L, 2203 aa. The 3’ leader of Cheonsu1510 was 55 nt and was highly homologous to those of known APMV serotypes. The first 12 nt of the leader sequence (3’-UCUUUGUUUGGU-5’) were identical to those of APMV-9. The 5’ trailers of AMPVs had variable sizes (17–776 nt); the Cheonsu1510 5’ trailer was 46 nt and was most closely related to those of APMV-9 and -15(Kr), both of which are 47 nt (Fig. 1). The first 12 nt of the 3’ leader and 5’ trailer sequences (5’-AGAAACAAACCA-3’) of Cheonsu1510 were perfectly complementary to each other. The gene-start (GS) and gene-end (GE) sequences of the six genes were 3’-UGCCCAUC(C)UU-5’ and 3’-AAUCU_6_-5’ respectively, and were well conserved. The P gene of Cheonsu1510 contained a putative RNA editing site (3’-UUUUUCCC-5’) at 2,272–2,281 in the genome, which relates to the production of additional V and W proteins through the insertion of single and double G residues, respectively [10]. The sequences were identical to those of most of the APMV groups, except for APMV-3 and -4 (3’-AAUUUCCC-5’), -11 (3’-UCUUAGUC-5’), and -14 (3’-AUUUUCCC-5’). The F protein cleavage site of Cheonsu1510 is D-R-E-G-R↓ L, which lacks multiple basic residues and resembles the aa motif of the lentogenic APMV-1. Comparison of the full genome sequences revealed that APMV-9 (63.0%), -15(Kr) (55.8%), -1 (55.7%), and -12 (51.9 %) are most closely related to Cheonsu1510, which shares less than 50% identity with representatives of other APMV groups (Online Resource 2).

**Fig. 1:**
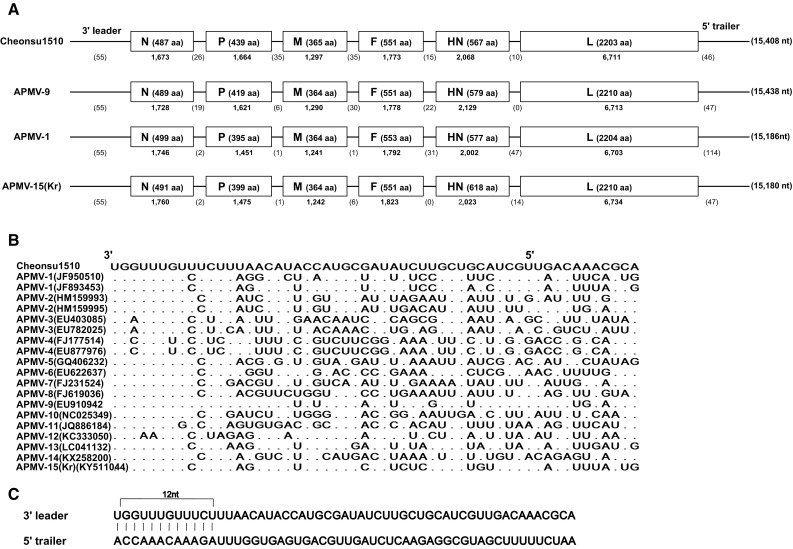
Genetic features of APMV/wild birds/Cheonsu1510/2015 (Cheonsu1510). **A.** Genetic map of Cheonsu1510, APMV-1, -9 and -15(Kr). Individual genes are indicated by rectangles. The amino acid length of each encoded protein is shown in each box, and the nucleotide (nt) length of each gene is shown under each box. The nt lengths of the non-coding leader, intergenic, and trailer regions are shown under each map, and the total genome nt length is shown in parentheses to the right. **B.** Sequence alignment (negative sense) of the 3’ leaders of Cheonsu1510 and other AMPV 1-15s. Dots indicate nucleotides identical to those in Cheonsu1510. **C.** Terminal complementarity between the 3’ leader and 5’ trailer of Cheonsu1510

As shown in Fig. 2A, a phylogenetic tree of complete genome sequences placed Cheonsu1510 on a major branch with APMV-1, -9, and -15(Kr), with a bootstrap value of 100. Within the group, Cheonsu1510 was closer to APMV-9 than APMV-1 and -15(Kr), consistent with the nt sequence identity results. The F and HN genes of Cheonsu1510 also clustered with APMV-1, -9, and -15(Kr), with a bootstrap value of 99, but clearly formed a separate phylogenetic group, especially from APMV-9 isolates from New York and Italy (Fig 2B and C). Consistently, when evolutionary distance from known serotypes of APMVs was calculated, Cheonsu1510 was relatively close to APMV-9 (0.453), -1 (0.586), and -15(Kr) (0.589; Online Resource 3).

**Fig. 2:**
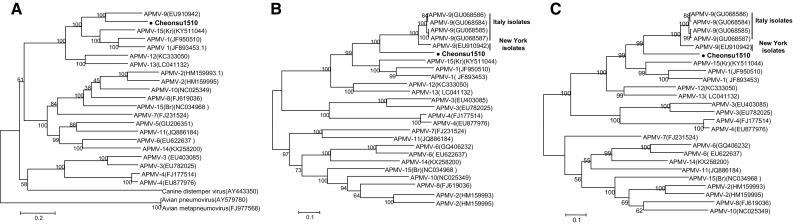
Phylogeny of APMV/wild birds/Cheonsu1510/2015 (Cheonsu1510). The phylogenetic distances of the full genomes (A), the F genes (B), and HN genes (C) of Cheonsu1510 and known AMPVs were calculated by the maximum-likelihood algorithm using MEGA 6, and trees were visualized using FigTree 1.3.1. Bootstrap values are shown as percentages of 1,000 replicates. Bootstrap values less than 50% are shown in the phylogenetic trees. The scale bars represent the number of substitutions per site. Nucleotide sequences from the present study are indicated in red

The genome of Cheonsu1510 had several features in common with those of other APMVs: the gene order and composition (3’-leader-N-P-M-F-HN-L-trailer 5’), 3’-leader homology, existence of GE and GS signals, complementation of 3’ and 5’ terminal sequences (12 nt), and the existence of putative RNA editing sites in the P gene (Fig. 1). In contrast, the Cheonsu1510 genome displayed several differences from known APMVs, such as the length of the complete sequence, the transcriptional units, the untranslated region and aa sequences of the six genes, and the GE, GS and RNA-editing sequences of the P gene (Online Resource 4). Cheonsu1510 also harbors a unique cleavage site within the F protein (D-R-E-G-R↓ L). In addition, sequence identity between Cheonsu1510 and other AMPVs was relatively low (< 50%), with the exception of APMV-9, -15(Kr), and -1 at 63.0%, 55.8, and 55.7%, respectively. However, these levels of identity between distinct APMVs are not uncommon (e.g., between APMV-1 and APMV-15(Kr) (64.9%) and between APMV-12 and APMV-13 (58.2%); Online Resource 3)).

The biological and evolutionary characteristics of Cheonsu1510 also differ from those of known APMVs. A cross-HI test showed that Cheonsu1510 is serologically distinct from other APMVs, although it did cross-react weakly with APMV-1 (*R* *=* 0.063) and -9 (*R* *=* 0.044). These values are lower than the cross-reactivity observed between the most closely related groups of viruses, such as APMV-1 and -12 (*R* *=* 0.125), APMV-9 and -15(Kr) (*R* *=* 0.125), and APMV-1 and -15(Kr) (*R* *=* 0.088) [7]. Evolutionary divergence values between Cheonsu1510 and other AMPVs (0.453 with APMV-9 and 0.586 with APMV-1) were larger than the reported intra-serotypic distances of APMV-2 (0.291), -3 (0.292), and -6 (0.265) [7, 14]. These results indicate that Cheonsu1510 s evolved from a common ancestor of APMV-1, -9, and -15(Kr) and is now placed a distinct branch among the APMV groups.

In summary, the results obtained in the present study indicate that the Cheonsu1510 isolate is sufficiently serologically and genetically different from known APMVs for it to be considered the prototype of a new APMV group, APMV-17.

## Electronic supplementary material

Below is the link to the electronic supplementary material.

Supplementary material 1 (DOC 145 kb)
