# Supplementary material for: Complete genome sequence of a novel avian paramyxovirus isolated from wild birds in South Korea
Source: Arch Virol. 2017 Oct 16;163(1):223–7. doi: 10.1007/s00705-017-3588-6 (PMC5756290; doi:10.1007/s00705-017-3588-6)
Supplement: Supplementary file 1 — Supplementary material 1 (DOC 145 kb) [file 705_2017_3588_MOESM1_ESM.doc]

Online Resource 1. Antigenic relatedness among Cheonsu1510 and APMVs determined by Archetti and Horsfall calculations based on HI test results (Table 1)

| Virus | Antiserum to | | | | | | | | |
| --- | --- | --- | --- | --- | --- | --- | --- | --- | --- |
|  | Cheonsu1510 | APMV-1 | APMV-2 | APMV-3 | APMV-4 | APMV-6 | APMV-7 | APMV-8 | APMV-9 |
| Cheonsu1510 | 1.000 | 0.063 | 0.008 | 0.016 | 0.022 | 0.008 | 0.016 | 0.016 | 0.044 |
| APMV-1 |  | 1.000 | 0.003 | 0.031 | 0.031 | 0.004 | 0.022 | 0.008 | 0.031 |
| APMV-2 |  |  | 1.000 | 0.011 | 0.022 | 0.044 | 0.022 | 0.016 | 0.006 |
| APMV-3 |  |  |  | 1.000 | 0.016 | 0.011 | 0.044 | 0.022 | 0.022 |
| APMV-4 |  |  |  |  | 1.000 | 0.031 | 0.044 | 0.016 | 0.031 |
| APMV-6 |  |  |  |  |  | 1.000 | 0.022 | 0.011 | 0.006 |
| APMV-7 |  |  |  |  |  |  | 1.000 | 0.022 | 0.022 |
| APMV-8 |  |  |  |  |  |  |  | 1.000 | 0.008 |
| APMV-9 |  |  |  |  |  |  |  |  | 1.000 |

The antigenic relatedness (R) between the two viruses was calculated using the method of Archetti and Horsfall (1951): R = (r_1_ × r_2_)^1/2^, where r_1_ is the ratio of the heterologous HI titer to the homologous HI titer for virus 1 and r_2_ is the ratio of the heterologous HI titer to the homologous HI titer for virus 2.

Online Resource 2. Nucleotides sequence identities (%) among the complete isolates of Cheonsu 1510 and other AMPVs

| Virus | APMV-1 | APMV-2 | APMV-3 | APMV-4 | APMV-5 | APMV-6 | APMV-7 | APMV-8 | APMV-9 | APMV-10 | APMV-11 | APMV-12 | APMV-13 | APMV-14 | APMV-15(Br) | APMV-15(Kr) |
| --- | --- | --- | --- | --- | --- | --- | --- | --- | --- | --- | --- | --- | --- | --- | --- | --- |
| APMV-1 | ID |  |  |  |  |  |  |  |  |  |  |  |  |  |  |  |
| APMV-2 | 40.6 | ID |  |  |  |  |  |  |  |  |  |  |  |  |  |  |
| APMV-3 | 38.0 | 37.2 | ID |  |  |  |  |  |  |  |  |  |  |  |  |  |
| APMV-4 | 39.3 | 38.9 | 41.1 | ID |  |  |  |  |  |  |  |  |  |  |  |  |
| APMV-5 | 37.3 | 40.8 | 37.5 | 35.7 | ID |  |  |  |  |  |  |  |  |  |  |  |
| APMV-6 | 39.5 | 43.3 | 37.1 | 38.3 | 44.3 | ID |  |  |  |  |  |  |  |  |  |  |
| APMV-7 | 40.6 | 46.2 | 38.8 | 38.9 | 42.0 | 44.2 | ID |  |  |  |  |  |  |  |  |  |
| APMV-8 | 40.8 | 57.1 | 38.6 | 38.7 | 41.8 | 44.4 | 47.7 | ID |  |  |  |  |  |  |  |  |
| APMV-9 | 56.3 | 40.2 | 37.9 | 38.9 | 37.4 | 39.1 | 40.3 | 41.0 | ID |  |  |  |  |  |  |  |
| APMV-10 | 40.6 | 55.4 | 38.2 | 39.3 | 42.1 | 45.0 | 47.6 | 56.0 | 41.1 | ID |  |  |  |  |  |  |
| APMV-11 | 37.1 | 41.1 | 38.1 | 35.6 | 51.2 | 41.3 | 42.2 | 42.4 | 37.9 | 42.7 | ID |  |  |  |  |  |
| APMV-12 | 53.3 | 40.2 | 37.9 | 39.5 | 37.7 | 39.6 | 40.9 | 41.0 | 52.4 | 40.7 | 37.5 | ID |  |  |  |  |
| APMV-13 | 50.5 | 38.6 | 39.6 | 37.6 | 37.8 | 38.4 | 40.0 | 39.8 | 49.8 | 39.5 | 38.2 | 58.2 | ID |  |  |  |
| APMV-14 | 39.7 | 43.9 | 37.9 | 39.0 | 44.3 | 51.9 | 44.4 | 45.9 | 40.0 | 45.7 | 41.2 | 40.1 | 38.6 | ID |  |  |
| APMV-15(Br) | 40.1 | 53.1 | 37.4 | 39.2 | 40.7 | 43.7 | 45.9 | 53.3 | 40.4 | 54.3 | 41.7 | 40.5 | 38.5 | 44.3 | ID |  |
| APMV-15(Kr) | 64.9 | 40.7 | 37.6 | 39.0 | 37.0 | 39.1 | 40.8 | 41.1 | 56.3 | 40.5 | 37.3 | 53.1 | 50.9 | 39.9 | 40.6 | ID |
| Cheonsu1510 | 55.7 | 39.9 | 37.1 | 38.4 | 36.9 | 39.0 | 39.6 | 40.0 | 63.0 | 39.8 | 36.8 | 51.9 | 48.8 | 39.3 | 39.5 | 55.8 |

Online Resource 3. Estimates of evolutionary divergence among the complete isolates of Cheonsu 1510 and other AMPVs

| Virus   | APMV-1 | APMV-2 | APMV-3 | APMV-4 | APMV-5 | APMV-6 | APMV-7 | APMV-8 | APMV-9 | APMV-10 | APMV-11 | APMV-12 | APMV-13 | APMV-14 | APMV-15(Br) | APMV-15(Kr) |  | | --- | --- | --- | --- | --- | --- | --- | --- | --- | --- | --- | --- | --- | --- | --- | --- | --- | | APMV-1 |  |  |  |  |  |  |  |  |  |  |  |  |  |  |  |  | | APMV-2 | 0.961 |  |  |  |  |  |  |  |  |  |  |  |  |  |  |  | | APMV-3 | 1.038 | 1.050 |  |  |  |  |  |  |  |  |  |  |  |  |  |  | | APMV-4 | 1.064 | 1.060 | 0.913 |  |  |  |  |  |  |  |  |  |  |  |  |  | | APMV-5 | 0.977 | 0.827 | 1.042 | 1.058 |  |  |  |  |  |  |  |  |  |  |  |  | | APMV-6 | 0.960 | 0.821 | 1.018 | 1.044 | 0.708 |  |  |  |  |  |  |  |  |  |  |  | | APMV-7 | 0.980 | 0.822 | 1.018 | 1.075 | 0.829 | 0.826 |  |  |  |  |  |  |  |  |  |  | | APMV-8 | 0.964 | 0.561 | 1.015 | 1.078 | 0.824 | 0.822 | 0.792 |  |  |  |  |  |  |  |  |  | | APMV-9 | 0.566 | 0.962 | 1.046 | 1.072 | 0.989 | 0.981 | 0.985 | 0.944 |  |  |  |  |  |  |  |  | | APMV-10 | 0.962 | 0.567 | 1.027 | 1.062 | 0.822 | 0.795 | 0.797 | 0.574 | 0.951 |  |  |  |  |  |  |  | | APMV-11 | 0.977 | 0.797 | 1.011 | 1.037 | 0.685 | 0.798 | 0.799 | 0.788 | 0.960 | 0.778 |  |  |  |  |  |  | | APMV-12 | 0.637 | 0.972 | 1.048 | 1.056 | 0.975 | 0.960 | 0.979 | 0.952 | 0.661 | 0.969 | 0.972 |  |  |  |  |  | | APMV-13 | 0.644 | 0.982 | 1.019 | 1.062 | 0.995 | 0.951 | 0.964 | 0.948 | 0.667 | 0.969 | 0.979 | 0.494 |  |  |  |  | | APMV-14 | 0.988 | 0.856 | 1.020 | 1.050 | 0.732 | 0.633 | 0.855 | 0.816 | 0.982 | 0.820 | 0.827 | 0.981 | 0.982 |  |  |  | | APMV-15(Br) | 0.973 | 0.607 | 1.023 | 1.067 | 0.834 | 0.810 | 0.817 | 0.603 | 0.965 | 0.579 | 0.784 | 0.961 | 0.969 | 0.828 |  |  | | APMV-15(Kr) | 0.426 | 0.960 | 1.053 | 1.074 | 0.998 | 0.973 | 0.968 | 0.945 | 0.571 | 0.970 | 0.963 | 0.644 | 0.630 | 0.977 | 0.954 |  | | Cheonsu1510 | 0.586 | 0.979 | 1.073 | 1.083 | 1.017 | 0.981 | 1.008 | 0.982 | 0.453 | 1.000 | 0.997 | 0.672 | 0.689 | 1.000 | 1.003 | 0.589 | |
| --- | --- | --- | --- | --- | --- | --- | --- | --- | --- | --- | --- | --- | --- | --- | --- | --- | --- | --- | --- | --- | --- | --- | --- | --- | --- | --- | --- | --- | --- | --- | --- | --- | --- | --- | --- | --- | --- | --- | --- | --- | --- | --- | --- | --- | --- | --- | --- | --- | --- | --- | --- | --- | --- | --- | --- | --- | --- | --- | --- | --- | --- | --- | --- | --- | --- | --- | --- | --- | --- | --- | --- | --- | --- | --- | --- | --- | --- | --- | --- | --- | --- | --- | --- | --- | --- | --- | --- | --- | --- | --- | --- | --- | --- | --- | --- | --- | --- | --- | --- | --- | --- | --- | --- | --- | --- | --- | --- | --- | --- | --- | --- | --- | --- | --- | --- | --- | --- | --- | --- | --- | --- | --- | --- | --- | --- | --- | --- | --- | --- | --- | --- | --- | --- | --- | --- | --- | --- | --- | --- | --- | --- | --- | --- | --- | --- | --- | --- | --- | --- | --- | --- | --- | --- | --- | --- | --- | --- | --- | --- | --- | --- | --- | --- | --- | --- | --- | --- | --- | --- | --- | --- | --- | --- | --- | --- | --- | --- | --- | --- | --- | --- | --- | --- | --- | --- | --- | --- | --- | --- | --- | --- | --- | --- | --- | --- | --- | --- | --- | --- | --- | --- | --- | --- | --- | --- | --- | --- | --- | --- | --- | --- | --- | --- | --- | --- | --- | --- | --- | --- | --- | --- | --- | --- | --- | --- | --- | --- | --- | --- | --- | --- | --- | --- | --- | --- | --- | --- | --- | --- | --- | --- | --- | --- | --- | --- | --- | --- | --- | --- | --- | --- | --- | --- | --- | --- | --- | --- | --- | --- | --- | --- | --- | --- | --- | --- | --- | --- | --- | --- | --- | --- | --- | --- | --- | --- | --- | --- | --- | --- | --- | --- | --- | --- | --- | --- | --- | --- | --- | --- | --- | --- | --- | --- | --- | --- | --- | --- | --- | --- | --- | --- | --- | --- | --- | --- | --- |
|  |
|  |
| The number of base substitutions per site from between sequences are shown. Analyses were conducted using the Maximum Composite Likelihood model. The analysis involved 17 nucleotide sequences. Codon positions included were 1st+2nd+3rd+Noncoding. All positions containing gaps and missing data were eliminated. There were a total of 12925 positions in the final dataset. Evolutionary analyses were conducted in MEGA6. |
|  |
|  |

Online Resource 4. Genetic features of Cheonsu 1510 and representative APMVs

| Virus | F_0_ cleavage | Length (nt) | Nucleotides identity | Gene Starts(GS) sequence | Gene Ends(GE) sequence | RNA editing site | Genbank ID |
| --- | --- | --- | --- | --- | --- | --- | --- |
| Cheonsu 1510 | D**R**EG**R**↓ L | 15,408 | - | UGCCCAUC(C)UU | AAUCU_6_ | UUUUUCCC | MF594598 |
| APMV-1(lento_genic) | G**R**QG**R**↓ L | 15,186 | 55.7 | UGCCCAUCNU | AAUCU_6-7_ | UUUUUCCC | JF950510 |
| APMV-2/Chicken/England/7702/06 | **K**PAS**R**↓ F | 14,904 | 39.9 | CCCCCGCUGU | AAUUCU_6_ | UUUUUCCC | HM159993 |
| APMV-3/turkey/Wisconsin/68 | **R**PSG**R**↓ L | 16,182 | 37.1 | UCC(U/C)CGCCUU | AA(U/A)U(A/U)U_6_ | AAUUUCCC | EU782025 |
| APMV-4/KR/YJ/06 | DIQP**R**↓ F | 15,054 | 38.4 | CAC_3-4_UUCC | AAUUAAU_4-5_ | AAUUUCCC | EU877976 |
| APMV-5/budgerigar/Japan/Kunitachi/74 | **KRKKR**↓ F | 17,262 | 36.9 | CCCCCUUNNN | AAU(A/U)NU_5_ | UUUUUCCC | GU206351 |
| APMV-6/duck/Hong Kong/D199/77 | APEP**R**↓ L | 16,236 | 39.0 | CUC_5-6_UUC | AAUN_1-2_AU_4-6_ | UUUUUCCC | EU622637 |
| APMV-7/dove/Tennessee/4/75 | LPSS**R**↓ L | 15,480 | 39.6 | CUCCCNCUNN | AAUNNUUUNU_1-3_ | UUUUUCCC | FJ231524 |
| APMV-8/goose/Delaware/1053/76 | YPQT**R**↓ L | 15,342 | 40.0 | CCCCCGCUGG | AAUUCU_6_ | UUUUUCCC | FJ619036 |
| APMV-9/duck/New York/22/78 | I**R**EG**R**↓ I | 15,438 | 63.0 | UGCCCAUCUU | AAUNU_6_ | UUUUUCCC | EU910942 |
| APMV-10/penguin/Falkland Islands/324/07 | **K**PSQ**R**↓ I | 15,456 | 39.8 | UCCCCGCUGN | AAUNNU_5-6_ | UUUUUCCC | NC025349 |
| APMV-11/common_snipe/France/100212/2010 | SGT**KR**↓ F | 17,412 | 36.8 | CGCCCGCUUN | AAUNNU_6_ | UCUUAGUC | JQ886184 |
| APMV-12/widgeon/Italy/39201/05 | GRE**PR**↓ L | 15,312 | 51.9 | UGCCCGUCUU | AAUNNUUNU_3-4_ | UUUUUCCC | KC333050 |
| APMV-13/Shimane/67/00 | V**R**EN**R**↓ L | 16,146 | 48.8 | UGCCCGUCUU | AAUUCU_5-6_ | UUUUUCCC | LC041132 |
| APMV-14/duck/Japan/11OG0352/2011 | T**R**EG**K**↓ L | 15,444 | 39.3 | CUCCCCCNNN | AAUNNNU_3_NU_1-2_ | AUUUUCCC | KX258200 |
| APMV-15/WB/Kr/UPO216/2014 | LVQA**R**↓ L | 15,180 | 55.8 | UGCCCNUCUU | AAUNNU_5-6_ | UUUUUCCC | KY511044 |
